# Deficiency of sphingomyelin synthase 1 but not sphingomyelin synthase 2 reduces bone formation due to impaired osteoblast differentiation

**DOI:** 10.1186/s10020-019-0123-0

**Published:** 2019-12-17

**Authors:** Goichi Matsumoto, Chieko Hashizume, Ken Watanabe, Makoto Taniguchi, Toshiro Okazaki

**Affiliations:** 1 Department of Oral and Maxillofacial Surgery, Kanazawa Medical University, 1-1 Daigaku, Uchinada, Ishikawa 920-0293 Japan; 2 Department of Medicine, Division of General and Digestive Surgery, Kanazawa Medical University, Ishikawa, Japan; 3 Department of Bone and Joint Disease, National Center for Geriatrics and Gerontology, Aichi, Japan; 4 Department of Life Science, Medical Research Institute, Kanazawa Medical University, Ishikawa, Japan; 5 Research Institute for Bioresources and Biotechnology, Ishikawa Prefectural University, Ishikawa, Japan

**Keywords:** Sphingomyelin synthase 1, Sphingomyelin synthase 2, SP7, Osteoblast differentiation, Bone morphogenic protein 2, Smad1/5/8, p38

## Abstract

**Background:**

There are two isoforms of sphingomyelin synthase (SMS): SMS1 and SMS2. SMS1 is located in the Golgi apparatus only while SMS2 is located in both the plasma membrane and the Golgi apparatus. SMS1 and SMS2 act similarly to generate sphingomyelin (SM). We have undertaken the experiments reported here on SMS and osteoblast differentiation in order to better understand the role SMS plays in skeletal development.

**Methods:**

We analyzed the phenotype of a conditional knockout mouse, which was generated by mating a Sp7 promoter-driven Cre-expressing mouse with an SMS1-floxed SMS2-deficient mouse (*Sp7-Cre;SMS1*^*f/f*^*;SMS2*^*−/−*^ mouse).

**Results:**

When we compared *Sp7-Cre;SMS1*^*f/f*^*;SMS2*^*−/−*^ mice with C57BL/6, SMS2-deficient mice (*SMS1*^*f/f*^*;SMS2*^*−/−*^) and SP7-Cre positive control mice (*Sp7-Cre, Sp7-Cre;SMS1*^*+/+*^*;SMS2*^*+/−*^ and *Sp7-Cre;SMS1*^*+/+*^*;SMS2*^*−/−*^), we found that although cartilage formation is normal, *Sp7-Cre;SMS1*^*f/f*^*;SMS2*^*−/−*^ mice showed reduced trabecular and cortical bone mass, had lower bone mineral density, and had a slower mineral apposition rate than control mice. Next, we have used a tamoxifen-inducible knockout system in vitro to show that SMS1 plays an important role in osteoblast differentiation. We cultured osteoblasts derived from *ERT2-Cre;SMS1*^*f/f*^ *SMS2*^*−/−*^ mice. We observed impaired differentiation of these cells in response to Smad1/5/8 and p38 that were induced by bone morphogenic protein 2 (BMP2). However, Erk1/2 phosphorylation was unaffected by inactivation of SMS1.

**Conclusions:**

These findings provide the first genetic evidence that SMS1 plays a role in bone development by regulating osteoblast development in cooperation with BMP2 signaling. Thus, SMS1 acts as an endogenous signaling component necessary for bone formation.

## Introduction

Skeletal homeostasis requires a precise balance between bone-forming osteoblasts and bone-resorbing osteoclasts. Osteoblasts differentiate and produce the bone matrix during skeletal development (Ducy et al. 2000). Osteoblast differentiation is regulated by various transcriptional factors, such as runt-related transcription factor-2 (Runx2) and Sp7 (osterix), which have been identified as osteoblast lineage regulators (Stains and Civitelli 2003). Much progress has been made in understanding osteoblast differentiation, bone development, and the roles of bone morphogenetic protein (BMP), fibroblast growth factor (FGF), and the Wnt and JAK/STAT signaling pathways (Long 2011; Long and Ornitz 2013; Raisz 2005). However, the underlying molecular mechanisms of osteoblast differentiation remain unclear. Sphingolipids are important components of mammalian cell membranes, and sphingomyelin (SM) is a major subspecies (Hannun and Obeid 2002; Merrill Jr. 2002; Spiegel and Milstien 2002). Sphingolipids are integral parts of various lipid membranes and serve as important second messengers for signal transduction pathways affecting cell growth and differentiation (Merrill Jr et al. 1997). Synthesis of SM from ceramide and phosphatidylcholine is mediated by the SM synthase (SMS) family members SMS1 and SMS2 (Huitema et al. 2004; Tafesse et al. 2006; Yamaoka et al. 2004). SMS1 is located in the Golgi apparatus and is responsible for bulk production of SM, whereas SMS2 is located in both the plasma membrane and Golgi apparatus (Jeckel et al. 1990; Villani et al. 2008; Yeang et al. 2008). Although enzymological differences between SMS1 and SMS2 are not clearly understood, SMS1 is known to be homeostatic, while SMS2 reacts to a diversity of stresses (Taniguchi and Okazaki 2014). Homozygous SMS1-mutant mice exhibit moderate neonatal lethality, reduced body weight, loss of fat-tissue mass, and growth deterioration, all of which suggest that they have metabolic abnormalities (Yano et al. 2011). In contrast, homozygous SMS2-mutant mice do not exhibit overt physical abnormalities and show normal growth to adulthood. SMS2 deficiency inhibits induction of colitis-associated colon cancer (Ohnishi et al. 2017), ameliorates obesity induced by a high-fat diet (Li et al. 2011), attenuates LPS-induced lung injury (Gowda et al. 2011), reduces expression of drug transporters in the brain (Zhang et al. 2011), and reduces atherosclerosis (Liu et al. 2009).

SM is hydrolyzed into ceramide and phosphatidylcholine by sphingomyelinases (Jung et al. 2000), which are classified into three categories: acidic, alkaline, and neutral (Nilsson and Duan 1999; Stoffel 1999). So far, no skeletal abnormalities in mice lacking SMPD1 or SMPD2 activity have been reported. SMPD3 encodes neutral sphingomyelinase 2, a membrane-bound enzyme whose expression is restricted to bone, cartilage, and brain (Khavandgar et al. 2011). Of the two SMPD3-deficient mouse models that have been established, one carries a chemically induced deletion of *fro* in the *Smpd3* locus (*fro/fro* model) and the other was generated by gene targeting (*SMPD3*^*−/−*^ model) (Aubin et al. 2005; Guenet 1982; Stoffel et al. 2005). Both *fro/fro* and *SMPD3*^*−/−*^ mice show severe bone and tooth mineralization defects as well as gross skeletal abnormalities. *Fro/fro* mice have been shown to have reduced ceramide levels in their skeletal tissues (Aubin et al. 2005).

Sphingolipids have been identified as growth-regulating molecules which regulate skeletal development and tissue homeostasis. The roles that SMS1 and SMS2 play in SM production and skeletal development are incompletely understood. SM is involved in matrix mineralization as well as signaling of extracellular stimuli. To clarify the role of SM in osteoblast biology, the genes essential to SM biosynthesis, SMS1 and SMS2, were knocked out in the osteoblast lineage. SMS1 and SMS2 are thought to play different roles in SM synthesis: SMS1 is a constitutive SM synthase in the Golgi apparatus and SMS2 is a more regulated synthase on the plasma membrane. Not only constitutive SMS1 but also SMS2 genes are highly expressed in the osteoblast lineage. As a limitation of this study, it is still unclear whether SMS gene knock-out has an impact on the SM levels of osteoblasts in SMS-mutant mice. In this study we focused on how the SM-synthesis pathway affects bone morphogenesis and bone metabolism, and we determined the phenotype of *Sp7-Cre;SMS1*^*f/f*^ *SMS2*^*−/−*^ mice.

New conditional SMS2 knockout mice were generated by crossing Sp7 promotor-driven Cre-recombinase-expressing mice with mice homozygous for the floxed *SMS1* gene. These *Sp7-Cre;SMS1*^*f/f*^*;SMS2*^*−/−*^ mice exhibit delayed ossification and osteoblast dysfunction, whereas *SMS1*^*f/f*^*;SMS2*^*−/−*^ mice show normal skeletal development similar to that of C57BL/6 mice. To clarify the role of SMS1 in osteogenesis, we also established an in vitro tamoxifen-inducible SMS1 inactivation system in osteoblasts using mice expressing a Cre-recombinase estrogen receptor (ERT2-Cre) fusion protein. Mutation of SMS1 was found, for the first time, to play an important role in osteoblast differentiation and mineralization stimulated by BMP2. Furthermore, mutation of SMS1 was shown to inhibit BMP2-induced Smad and MAPK signaling in osteoblasts.

## Materials and methods

### Animal studies

Animals were housed in a temperature-controlled room with a 12-h light/12-h dark cycle. Food and water were available ad libitum unless noted. All experimental protocols were approved by the Kanazawa Medical University Ethics Review Committee for Animal Experimentation. To generate *SMS1*^*f/f*^*;SMS2*^*−/−*^ mice, *SMS1*^*f/f*^ mice, in which *SMS1* was flanked by *loxP* sequences, were crossed with *SMS2*^*−/−*^ mice (Ohnishi et al. 2017). Sp7-green fluorescent protein/Cre mice were purchased from Jackson Laboratories (stock no. 006361; Bar Harbor, ME, USA). These two strains were crossed and maintained on a C57BL/6 J background. After generating triple heterozygous *Sp7-Cre;SMS1*^*f/f*^*;SMS2*^*+/−*^ mice, they were bred with *SMS1*^*f/f*^*;SMS2*^*−/−*^ mice to generate SMS1 conditional knockout mice (*Sp7-Cre;SMS1*^*f/f*^*;SMS2*^*−/−*^) and Cre-null (*SMS1*^*f/f*^*;SMS2*^*−/−*^) mice. To establish conditional whole-body knockout mice, *SMS1*^*f/f*^*;SMS2*^*−/−*^ mice were crossed with ERT2-Cre mice (stock no: 008463; Jackson Laboratories) to enable temporal control of floxed gene expression by tamoxifen induction. *ERT2-Cre;SMS1* ^*f/f*^*;SMS2*^*−/−*^ mice were used for isolation and culture of primary calvarial osteoblasts. For genotyping, genomic DNA was isolated from mouse tails. Polymerase chain reactions (PCRs) were performed using a PCR Master Mix (Takara Bio, Inc., Shiga, Japan) with primer sequences for *Cre* transgene and floxed *SMS1* genes relative to wild-type *SMS1*.

### Skeletal preparation

Embryonic day 15.5 mice (E15.5), 17.5 mice (E17.5) and newborn mice were eviscerated, fixed in 100% ethanol for 4 days and then transferred to acetone. After 3 days, specimens were rinsed with water and stained for 10 days in starting solution consisting of 1 volume of 0.1% Alizarin Red S (for bone) in 95% ethanol, 1 volume of 0.3% Alcian blue 8GX (for cartilage) in 70% ethanol, 1 volume of 100% acetic acid, and 17 volumes of ethanol. After rinsing with 100% ethanol, specimens were maintained in 1% KOH at 37 °C for 24 h and then maintained in 20% glycerol/1% KOH at room temperature until the skeletons became clearly visible. For storage, specimens were transferred into 50, 80, and 100% glycerol (Komori et al. 1997).

### Micro- and peripheral quantitative (pQ)-computed tomography (CT)

We killed 15-week-old mice, and their femurs were fixed for 5 days in 100% ethanol before analyses by micro-CT (MCT-CB130F; Hitachi Medico, Tokyo, Japan) and pQ-CT (XCT Research SA+; Stratec Medizintechnik, Germany). We scanned femurs according to previously published procedures (Matsumoto et al. 2012).

### Histological and histomorphometric analyses

The bones of 15-week-old mice were fixed at 4 °C overnight in 4% paraformaldehyde and then decalcified with 10% EDTA for 10 days. Paraffin-embedded femurs were sectioned (5-μm thick) and stained with hematoxylin and eosin (H&E). The overall width of the growth plate was measured from the metaphyseal margin of the terminal hypertrophic cell lacuna to the epiphyseal margin of the resting zone. Measurements were made as previously described (Marino et al. 2008). This measurement was performed in three areas of the growth plate and averaged. Non-decalcified, paraffin-embedded femur sections were evaluated by von Kossa staining using standard methods. Calcein double-labeling of femurs in mice was performed with calcein injections (16 mg·kg^− 1^ body weight) at 3-day intervals.

For histomorphometric analysis, femurs were excised, fixed with 75% ethanol, embedded in glycomethacrylate resin, and sectioned (3-μm thick). Sections were stained with toluidine blue and histomorphometrically analyzed under light microscopy with a micrometer, using a semi-automated image-analysis system (OsteoMeasure; OsteoMetrics, Atlanta, GA, USA). Parameters of the trabecular bone were measured in an area 1.28–2.09 mm^2^ in size from 1.125 mm above the growth plate at the distal metaphysis.

### Quantitative RT-PCR (qRT-PCR) analysis

Total RNA and cDNA were prepared by ISOGEN® (Wako, Osaka, Japan) and ReverTra Ace quantitative PCR Real-Time Master Mix (Toyobo, Osaka, Japan) according to the manufacturer’s instructions. We performed qRT-PCR with a QuantStudio 12 K Flex Real-Time PCR System (Thermo Fisher Scientific, Waltham, MA, USA) using TaqMan probes (Thermo Fisher Scientific) for collagen type I α1 (*COL1A1*; Mm00801666_g1), alkaline phosphatase (*ALP*; Mm00475834_m1), bone γ-carboxyglutamic acid (Gla) osteocalcin (OCN) protein (*OCN*; Mm03413826_mH), secreted phosphoprotein 1 (*OPN*; Mm00436767_m1), integrin binding sialoprotein (*BSP*; Mm00492555_m1), receptor activator of nuclear factor κB (*RANK*; Mm00435454_m1), tumor necrosis factor ligand superfamily, member 11 (*RANKL*; Mm00441908_m1), *SMS1* (Mm00522643_m1), *SMS2* (Mm00512327_m1), dentin matrix acidic phosphoprotein 1 (*DMP1*; Mm01208363_m1), phosphate-regulating endopeptidase homolog, X-linked (*PHEX*; Mm.2529), Osteoprotegerin (*OPG*; Mm00435454_m1) and TaqMan Master Mix (Thermo Fisher Scientific). Target gene expression was normalized to glyceraldehyde-3-phosphate dehydrogenase (*GAPDH*; Mm99999915_g1) expression.

### Isolation and culture of primary osteoblasts

Calvarial bones from newborn *ERT2-Cre;SMS1*^*f/f*^*;SMS2*^*−/−*^ and *SMS1*^*f/f*^*;SMS2*^*−/−*^ mice were collected by chipping with scissors. Pieces of bones were then digested twice with 0.1% collagenase type I (Wako Pure Chemical, Osaka, Japan) in phosphate-buffered saline at 37 °C on a shaker for 15 min. Subsequently, pieces of bones were digested three times in succession without pause with 0.1% collagenase for 20 min, and osteoblastic populations were obtained from each solution to give fractions 3, 4, and 5 (Suto et al. 2012). Osteoblastic populations were centrifuged at 1500 rpm for 5 min, resuspended in α-minimum essential medium (α-MEM) with 10% fetal bovine serum (FBS), 100 U/mL of penicillin, and 100 μg/mL of streptomycin. For cell-growth assays, osteoblasts were seeded on 24-well plates (IWAKI Cell Biology, Tokyo, Japan) at 2 × 10^4^ cells/well. The next day, osteoblasts were treated with 1 μM of 4-hydroxytamoxifen (4-OHT) for 24 h, treated with 5 ng of FGF2 (R&D Systems, Minneapolis, MN, USA), and allowed to proliferate for an additional 3 days. For differentiation assays, osteoblasts were seeded at a density of 2.5 × 10^4^ cells/cm^2^ on culture plates. Similar to the proliferation assay, the following day, osteoblasts were treated with 4-OHT and differentiation was induced by adding osteogenic medium (α-MEM containing 10% FBS, 10 mM β-glycerophosphate, 50 μg/mL ascorbic acid, and 100 ng/mL BMP2). ALP staining was performed using a tartrate-resistant acid phosphatase (TRAP)/ALP staining kit (Wako Pure Chemical). ALP activity was measured in cell lysates using a LabAssay™ ALP kit (Wako Pure Chemical). Gla-OCN synthesis was measured in culture medium using a mouse Gla-OCN competitive enzyme-linked immunosorbent assay kit (Takara Bio). To quantify terminal differentiation and mineralization of osteoblasts, cultured osteoblasts were fixed with phosphate-buffered formalin and then stained with 40 mM Alizarin Red S (pH 4.2; Sigma-Aldrich) for 30 min at room temperature. After washing with distilled water, the plates were photographed. Alizarin Red S dye was extracted with 10% formic acid, and the absorbance at 415 nm was determined with a microplate reader.

### Western blot analysis

Western blot analysis of cell extracts was performed as described previously (Ohnishi et al. 2017). Briefly, cells were washed with phosphate-buffered saline three times then lysed in RIPA buffer [50 mmol/L Tris-HCl (pH 8.0), 150 mmol/L NaCl, 0.5% (w/v) sodium deoxycholate, 0.1% (w/v) sodium dodecyl sulfate, and 1.0% (w/v) NP-40 substitute]. After incubation on ice for 20 min, debris was removed by centrifuging at 2000 *g* for 10 min at 4 °C. Supernatant proteins (20 μg) were electrophoresed with sodium dodecyl sulfate polyacrylamide gel and transferred to polyvinylidene fluoride membranes (Millipore, Billerica, MA, USA). Nonspecific binding was blocked by incubating membranes with Blocking One–P (Nacalai Tesque, Kyoto, Japan) for 20 min at room temperature. Then membranes were incubated overnight with primary antibodies, including anti-phospho-Smad1/5/8 (1:1000), anti-Smad1 (1:1000), anti-phospho-ERK1/2 (1:1000), and anti-ERK1/2 (1:1000), anti-phospho-p38 (1:1000), anti-p38 (1:1000) [all from Cell Signaling Technology, Danvers, MA, USA], at 4 °C and then with secondary antibodies for 45 min at room temperature. Immunoreactive protein bands were visualized using an ECL-peroxidase detection system (Thermo Fisher Scientific) and LAS-4000 (Fujifilm, Tokyo, Japan).

### Statistical analysis

The results are expressed as mean ± standard deviation. Statistical comparisons between experimental groups were carried out using Student’s *t*-test. A *P* < 0.05 was considered statistically significant.

## Results

### Osteoblast-specific deletion of SMS1 caused delayed ossification

**Fig. 1:**
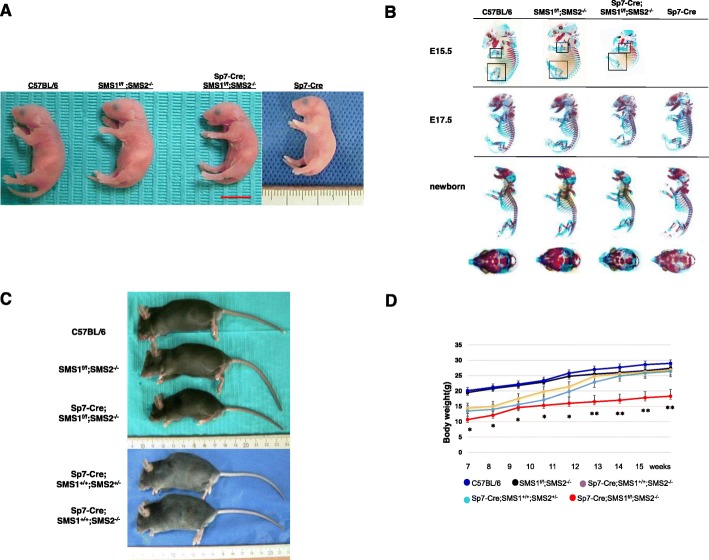
Skeletal changes and growth retardation in *Sp7-Cre;SMS1*^*f/f*^*;SMS2*^*−/−*^ mice. **a** Gross appearance of C57BL/6, *SMS1*^*f/f*^*;SMS2*^*−/−*^, *Sp7-Cre;SMS1*^*f/f*^*;SMS2*^*−/−*^ and *Sp7-Cre* mice at p1. *Sp7-Cre;SMS1*^*f/f*^*;SMS2*^*−/−*^ mice are small. Bar = 1 cm. **b** Skeletons from E15.5-E17.5 embryos and newborns (p1) of C57BL/6, *SMS1*^*f/f*^*;SMS2*^*−/−*^, *Sp7-Cre;SMS1*^*f/f*^*;SMS2*^*−/−*^ *and Sp7-Cre* mice. Embryos and newborns were stained with Alizarin red and Alcian blue. Note the delayed mineralization of the limbs in the *Sp7-Cre;SMS1*^*f/f*^*;SMS2*^*−/−*^ E15.5 embryo compared with that of C57BL/6 and *SMS1*^*f/f*^*;SMS2*^*−/−*^ mice (black square). Dorsal views of skulls of newborns showed that calvarial bone was hypomineralized in *Sp7-Cre;SMS1*^*f/f*^*;SMS2*^*−/−*^ mice. **c** Gross morphology of C57BL/6, *SMS1*^*f/f*^*;SMS2*^*−/−*^, *Sp7-Cre;SMS1*^*f/f*^*;SMS2*^*−/−*^*, Sp7-Cre;SMS1*^*+/+*^*;SMS2*^*+/−*^*,* and *Sp7-Cre;SMS1*^*+/+*^*;SMS2*^*−/−*^ male mice at 15 weeks after birth. **d** Body weight of C57BL/6, *SMS1*^*f/f*^*;SMS2*^*−/−*^, *Sp7-Cre;SMS1*^*f/f*^*;SMS2*^*−/−*^*, Sp7-Cre;SMS1*^*+/+*^*;SMS2*^*+/−*^*,* and *Sp7-Cre;SMS1*^*+/+*^*;SMS2*^*−/−*^ male mice at indicated time points (*n* = 10 for each genotype). *p < 0.05 versus C57BL/6 and *SMS1*^*f/f*^*;SMS2*^*−/−*^. **p < 0.05 versus C57BL/6, *SMS1*^*f/f*^*;SMS2*^*−/−*^, *Sp7-Cre;SMS1*^*+/+*^*;SMS2*^*−/−*^*,* and *Sp7-Cre;SMS1*^*+/+*^*;SMS2*^*+/−*^. Data are mean ± SD

First, we generated double knockout (*Sp7-Cre;SMS1*^*f/f*^*;SMS2*^*−/−*^) mice by crossing *Sp7* promoter-driven Cre-recombinase-expressing mice with *SMS1*-floxed *SMS2*^*−/−*^ mice (*SMS1*^*f/f*^*;SMS2*^*−/−*^). There are no statistical differences between the skeletal phenotype and body weight of *Sp7-Cre;SMS*^*+/f*^*;SMS2*^*+/−*^ mice compared to C57BL/6 mice (data not shown). Alizarin Red S and Alcian blue staining of skeletal preparations was performed for E15.5 and newborn C57BL/6, *SMS1*^*f/f*^*;SMS2*^*−/−*^, *Sp7-Cre* and *Sp7-Cre;SMS1*^*f/f*^*;SMS2*^*−/−*^ mice. Newborn, E17.5 and E15.5 *Sp7-Cre;SMS1*^*f/f*^*;SMS2*^*−/−*^ mice were smaller than littermate controls, but no significant patterning changes were observed in their bone or cartilage. In the early stages of ossification (E15.5), the calvaria and long limb bones of *Sp7-Cre;SMS1*^*f/f*^*;SMS2*^*−/−*^ mice showed poorer development, manifested by loosely mineralized bone structure and shorter long limb bones. At birth, mineralization of calvarial bone was delayed in *Sp7-Cre;SMS1*^*f/f*^*;SMS2*^*−/−*^ mice, but no skeletal defects were observed in *SMS1*^*f/f*^*;SMS2*^*−/−*^ and *Sp7-Cre* mice. Moreover, *Sp7-Cre;SMS1*^*f/f*^*;SMS2*^*−/−*^ newborns had less nasal bone development around the nasal capsule than did their littermate controls (Fig. 1a, b). No differences were observed in ossification between *Sp7-Cre, SMS1*^*f/f*^*;SMS2*^*−/−*^ and C57BL/6 mice. These results are indicative of a bone-specific deficiency of SMS1 in *Sp7-Cre;SMS1*^*f/f*^*;SMS2*^*−/−*^ mice. The effect of SMS1 and/or SMS2 deficiency on postnatal skeletal growth was evaluated in *Sp7-Cre;SMS1*^*f/f*^*;SMS2*^*−/−*^ mice relative to sex-matched littermate controls. As shown in Fig. 1c, d, the growth of *Sp7-Cre;SMS1*^*f/f*^*;SMS2*^*−/−*^ mice was retarded.

### Sp7-Cre;SMS1^f/f^;SMS2^−/−^ mice showed osteopenia

**Fig. 2:**
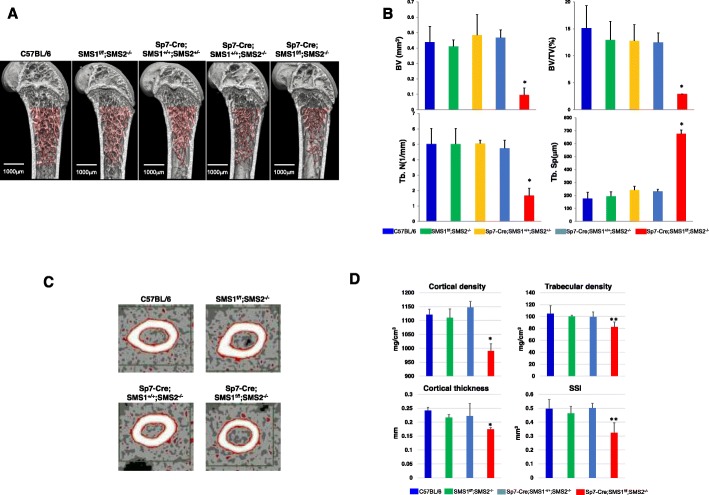
Note the osteoporotic phenotype of the *Sp7-Cre;SMS1*^*f/f*^*;SMS2*^*−/−*^ mice. **a** Representative micro-CT images of the distal femoral shafts of 15-week-old C57BL/6, *SMS1*^*f/f*^*;SMS2*^*−/−*^, *Sp7-Cre;SMS1*^*+/+*^*;SMS2*^*+/−*^, *Sp7-Cre;SMS1*^*+/+*^*;SMS2*^*−/−*^ and *Sp7-Cre;SMS1*^*f/f*^*;SMS2*^*−/−*^ mice. 3D reconstruction of trabecular bone. **b** Micro-CT analysis of femurs from 15-week-old mice. Abbreviations: BV, bone volume; BV/TV, bone volume/total volume; Tb N, trabecular number; Tb Sp, trabecular separation; *p < 0.05 versus C57BL/6, *SMS1*^*f/f*^*;SMS2*^*−/−*^, *Sp7-Cre;SMS1*^*+/+*^*;SMS2*^*+/−*^ and *Sp7-Cre;SMS1*^*+/+*^*;SMS2*^*−/−*^; mean ± SD, n = 10 for each genotype. **c** Isolated femurs of 15-week-old mice were scanned using pQCT. Sections were made at the midshaft. **d** Quantification of cortical density, trabecular density, cortical thickness and strength strain index (SSI). *p < 0.005 versus C57BL/6, *SMS1*^*f/f*^*;SMS2*^*−/−*^, and *Sp7-Cre;SMS1*^*+/+*^*;SMS2*^*−/−*^, **p < 0.05 versus C57BL/6, *SMS1*^*f/f*^*;SMS2*^*−/−*^ and *Sp7-Cre;SMS1*^*+/+*^*;SMS2*^*−/−*^; mean ± SD, *n* = 8–10 for each genotype

Micro-CT analysis revealed microstructural changes in femurs of *Sp7-Cre;SMS1*^*f/f*^*;SMS2*^*−/−*^ mice, where bone forms via endochondral ossification. Micro-CT analysis of femurs in 15-week-old *Sp7-Cre;SMS1*^*f/f*^*;SMS2*^*−/−*^ mice showed that trabecular bone parameters were markedly lower compared to C57BL/6, *SMS1*^*f/f*^*;SMS2*^*−/−*^, *Sp7-Cre;SMS1*^*+/+*^*;SMS2*^*+/−*^, and *Sp7-Cre;SMS1*^*+/+*^*;SMS2*^*−/−*^ mice. Compared with age-matched controls, the femurs of *Sp7-Cre;SMS1*^*f/f*^*;SMS2*^*−/−*^ mice had reduced bone-volume fraction (77–80% decrease) and decreased trabecular numbers (63–66% decrease) accompanied by greater trabecular separation (Fig. 2a, b). In contrast, *SMS1*^*f/f*^*;SMS2*^*−/−*^, *Sp7-Cre;SMS1*^*+/+*^*;SMS2*^*+/−*^, *Sp7-Cre;SMS1*^*+/+*^*;SMS2*^*−/−*^, and C57BL/6 mice showed no differences.

Next, we quantified cortical bone mineral density, cortical thickness, and the strength-strain index at the midshaft of femurs in 15-week-old mice by pQ-CT. These results revealed a reduced mineral density in both cortical and trabecular bones of *Sp7-Cre;SMS1*^*f/f*^*;SMS2*^*−/−*^ mice. Cortical thickness was also reduced by 30% in femurs of *Sp7-Cre;SMS1*^*f/f*^*;SMS2*^*−/−*^ mice versus that of controls. Bone strength was established with respect to torsion and found to be significantly lower at the distal femoral shaft of *Sp7-Cre;SMS1*^*f/f*^*;SMS2*^*−/−*^ mice than in controls (Fig. 2c, d). Collectively, these quantitative micro- and pQ-CT results agree with qualitative images, suggesting reduced trabecular and cortical bone mineralization in *Sp7-Cre;SMS1*^*f/f*^*;SMS2*^*−/−*^ mice. These results imply that skeletal abnormalities in *Sp7-Cre;SMS1*^*f/f*^*;SMS2*^*−/−*^ mice persist as these mice age.

### Mutation of SMS1 reduced bone mineralization rates

**Fig. 3:**
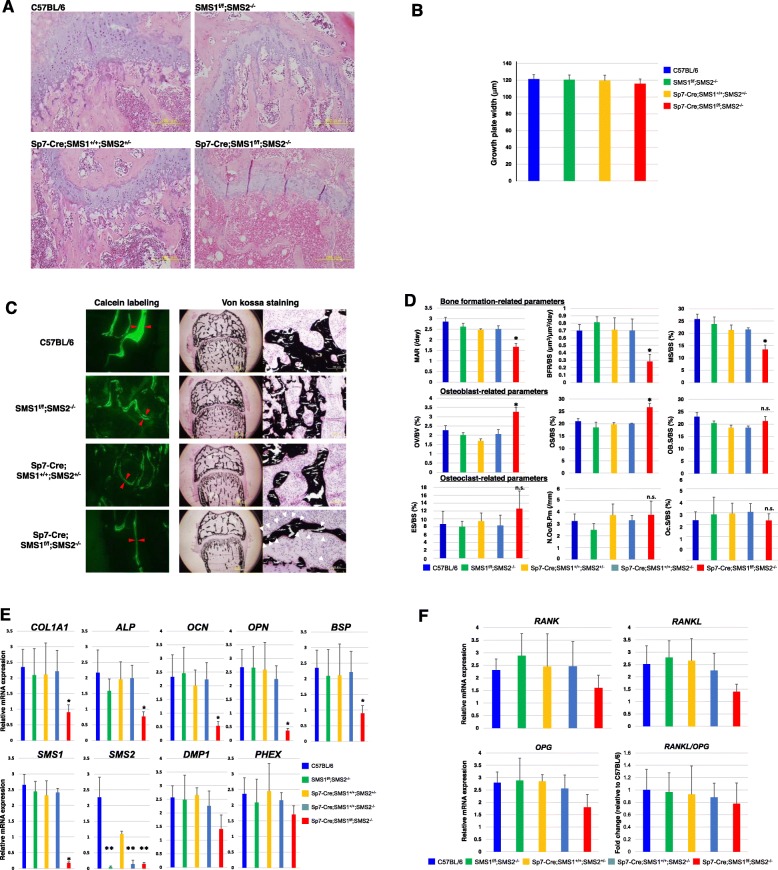
Decreased bone formation in adult *Sp7-Cre;SMS1*^*f/f*^*;SMS2*^*−/−*^ mice. **a** Hematoxylin-eosin staining of the distal femurs of 15-week-old C57BL/6, *SMS1*^*f/f*^*;SMS2*^*−/−*^, *Sp7-Cre;SMS1*^*+/+*^*;SMS2*^*+/−*^, and *Sp7-Cre;SMS1*^*f/f*^*;SMS2*^*−/−*^ mice (scale bar 200 μm). **b** Quantitative analysis of width of the growth plate in C57BL/6, *SMS1*^*f/f*^*;SMS2*^*−/−*^, *Sp7-Cre;SMS1*^*+/+*^*;SMS2*^*+/−*^, and *Sp7-Cre;SMS1*^*f/f*^*;SMS2*^*−/−*^ mice. **c** Calcein double-labeling at an interval of 3 days of femurs of 15-week-old C57BL/6, *SMS1*^*f/f*^*;SMS2*^*−/−*^, *Sp7-Cre;SMS1*^*+/+*^*;SMS2*^*+/−*^, and *Sp7-Cre;SMS1*^*f/f*^*;SMS2*^*−/−*^ mice. The distance between two calcein-labeled mineralization fronts is indicated by red arrow heads at the midshaft of femurs (left). Von Kossa staining of the distal femurs of 15-week-old C57BL/6, *SMS1*^*f/f*^*;SMS2*^*−/−*^, *Sp7-Cre;SMS1*^*+/+*^*;SMS2*^*+/−*^, and *Sp7-Cre;SMS1*^*f/f*^*;SMS2*^*−/−*^ mice. Undemineralized sections of the femurs of *Sp7-Cre;SMS1*^*f/f*^*;SMS2*^*−/−*^ mice show that there is little trabecular interconnection (middle), that bone mass is almost normal, and that there is extensive accumulation of osteoid (right). Mineralized bone is stained black, unmineralized osteoid is stained in red. Osteoid is indicated by white arrow heads. **d** Histomorphometric analysis of the femurs of 15-week-old C57BL/6, *SMS1*^*f/f*^*;SMS2*^*−/−*^, *Sp7-Cre;SMS1*^*+/+*^*;SMS2*^*+/−*^*, Sp7-Cre;SMS1*^*+/+*^*;SMS2*^*−/−*^, and *Sp7-Cre;SMS1*^*f/f*^*;SMS2*^*−/−*^ mice. MAR, mineral apposition rate; BFR/BS, bone formation rate/bone surface; MS/BS, mineralizing surface/bone surface; OV/OB, osteoid volume/bone volume; OS/BS, osteoid surface/bone surface; Ob.S/BS, Osteoblast surface/bone surface; ES/BS, eroded surface/bone surface; N.Oc/B.Pm, Osteoclast numbers/osteoclast perimeter; Oc.S/BS, osteoclast surface/bone surface; *, *p* < 0.05 versus C57BL/6, *SMS1*^*f/f*^*;SMS2*^*−/−*^, *Sp7-Cre;SMS1*^*+/+*^*;SMS2*^*+/−*^, and *Sp7-Cre;SMS1*^*+/+*^*;SMS2*^*−/−*^; mean ± SD, *n* = 8–10 for each genotype. **e** Relative abundance of gene expression of calvarial bone from C57BL/6, *SMS1*^*f/f*^*;SMS2*^*−/−*^, *Sp7-Cre;SMS1*^*+/+*^*;SMS2*^*+/−*^, *Sp7-Cre;SMS1*^*+/+*^*;SMS2*^*−/−*^, and *Sp7-Cre;SMS1*^*f/f*^*;SMS2*^*−/−*^ mice. Significant downregulation of COL1A1, ALP, OCN, OPN, and BSP expression was observed in Sp7-Cre;SMS1f/f;SMS2−/− mice. **f** Relative abundance of RANK, RANKL, and OPG expression and the RANKL/OPG ratio for each mouse. Quantitative analysis of RT-PCR using RNA from p5 calvarial bone. Mean ± SD, *p < 0.05 versus C57BL/6, *SMS1*^*f/f*^*;SMS2*^*−/−*^ and *Sp7-Cre;SMS1*^*+/+*^*;SMS2*^*+/−*^*,* **p < 0.005 versus C57BL/6 and *Sp7-Cre;SMS1*^*+/+*^*;SMS2*^*+/−*^; *n* = 10 for each genotype

H&E-stained sections of distal femurs revealed that trabecular bone mass near the growth plate of *Sp7-Cre;SMS1*^*f/f*^*;SMS2*^*−/−*^ mice was reduced than those of C57BL/6, *SMS1*^*f/f*^*;SMS2*^*−/−*^ and *Sp7-Cre;SMS1*^*+/+*^*;SMS2*^*+/−*^ mice. The epiphyseal growth plate and cartilaginous tissues of *Sp7-Cre;SMS1*^*+/+*^*;SMS2*^*+/−*^ and *Sp7-Cre;SMS1*^*f/f*^*,SMS2*^*−/−*^ mice were thinner than those of control mice, however, there was no significant difference (Fig. 3a, b).

To further clarify the effect of SMS1 deficiency on reduced bone mass and BMD, we compared in vivo mineral apposition in *Sp7-Cre;SMS1*^*f/f*^*;SMS2*^*−/−*^ and control mice via calcein double-labeling. A smaller distance between the calcein-labeled mineralization front at the midshaft of femurs was observed in *Sp7-Cre;SMS1*^*f/f*^*;SMS2*^*−/−*^ mice versus controls, suggesting less new bone formation in *Sp7-Cre;SMS1*^*f/f*^*;SMS2*^*−/−*^ mice. In undecalcified Von Kossa staining, the osteoid was stained red and mineralized bone substance appeared black. Greater osteoid formation was seen in *Sp7-Cre;SMS1*^*f/f*^*;SMS2*^*−/−*^ mice compared to control mice (Fig. 3c).

Histomorphometric analysis showed that the mineral apposition rate (MAR), bone formation rate (BFR/BS), and mineralized surface (MS/BS) of *Sp7-Cre;SMS1*^*f/f*^*;SMS2*^*−/−*^ mice were 25–40%, 40–55% and 30–40% lower, respectively, than those of controls. It also revealed that osteoblast-related parameters (osteoid volume/bone volume (OV/BV), osteoid surfaces (OS/BS), and osteoblast surfaces (ob. S/BS) were significantly increased in *Sp7-Cre;SMS1*^*f/f*^*;SMS2*^*−/−*^ mice compared to controls. In contrast, osteoclast-related parameters eroded surfaces (ES/BS), osteoclast numbers (N.Oc/B.Pm), and osteoclast surfaces (Oc.S/BS) were not affected (Fig. 3d). Combined with the micro- and pQ-CT data above, the results suggest that a lower bone mineralization rate and an increase in the number of osteoids contributed, at least in part, to the lower bone mass found in *Sp7-Cre;SMS1*^*f/f*^*;SMS2*^*−/−*^ mice. To gain further insight into the potential mechanisms underlying defective bone mineralization in *Sp7-Cre;SMS1*^*f/f*^*;SMS2*^*−/−*^ mice, we compared gene expression in femur bones of C57BL/6, *SMS1*^*f/f*^*;SMS2*^*−/−*^, *Sp7-Cre;SMS1*^*+/+*^*;SMS2*^*+/−*^, *Sp7-Cre;SMS1*^*+/+*^*;SMS2*^*−/−*^, and *Sp7-Cre;SMS1*^*f/f*^*;SMS2*^*−/−*^ mice at postnatal day 5 using qRT-PCR. The results showed that COL1A1, ALP, OCN, OPN, and BSP mRNA were downregulated significantly, while a mild downregulation of DMP1 and PHEX expression was observed (Fig. 3e). Next, we measured the gene expression of RANK, RANKL, and OPG, and we computed the RANKL/OPG ratio. Interestingly, RANK, RANKL, OPG, and the RANKL/OPG ratio were mildly downregulated but not statistically significantly in Sp7-Cre;SMS1f/f;SMS2−/− mice compared to control mice (Fig. 3f).

### Inactivation of SMS1-impaired BMP2-induced osteoblast differentiation

**Fig. 4:**
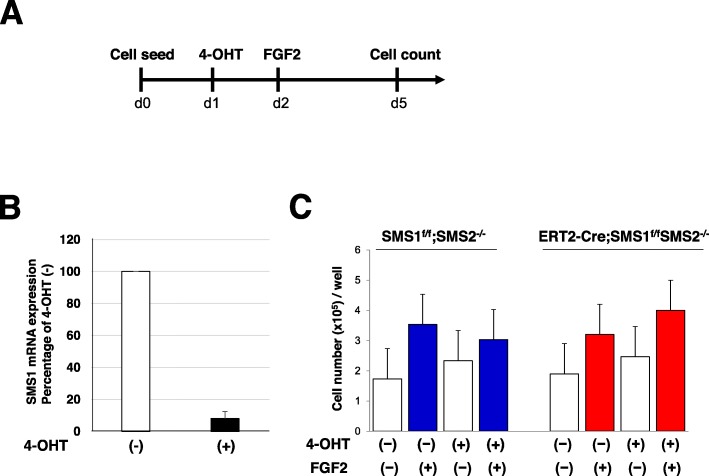
Effects of *SMS1* knock-out on osteoblast cell growth. **a** Schematic outline of the experimental design. Calvarial osteoblast cells were seeded on 48-well plates (5000 cells/well) with 10% FBS containing α-MEM. **b** Reduction of expression of SMS1 in osteoblasts treated with 1 μM 4-hydroxy tamoxifen (4-OHT). Primary osteoblast cells were isolated from the calvaria of newborn *ERT2-Cre;SMS1*^*f/f*^*;SMS2*^*−/−*^ mice. **c** The number of cells was counted 4 days after treatment with 4-OHT. Inactivation of SMS1 resulted in no growth inhibition of calvarial osteoblasts. Treatment with 4-OHT does not affect the cell growth of primary *SMS1*^*f/f*^*;SMS2*^*−/−*^ calvarial osteoblasts. Osteoblasts were prepared from newborn *SMS1*^*f/f*^*;SMS2*^*−/−*^ and *ERT2-Cre;SMS1*^*f/f*^*;SMS2*^*−/−*^ mice. Values are expressed as the mean ± SD, *n* = 8 for each genotype

**Fig. 5:**
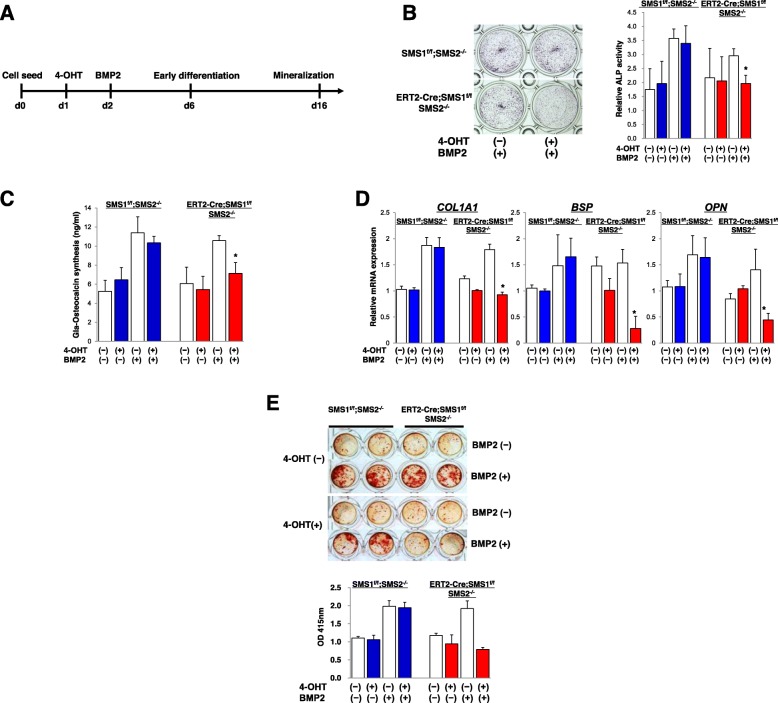
Effects of *SMS1* knock-out on osteoblast differentiation. **a** Schematic outline of the experimental design. **b** Representative pictures of ALP-staining of primary calvarial osteoblast cultures in the absence or presence of 1 μM 4-OHT at day 6 after BMP2 stimulation (left). Quantification of the staining is shown (right). Inactivation of *SMS1* resulted in reduced ALP activity of calvarial osteoblasts. *p < 0.005 versus *SMS1*^*f/f*^*;SMS2*^*−/−*^ (4-OHT+, BMP2+) and *ERT2-Cre;SMS1*^*f/f*^*;SMS2*^*−/−*^(4-OHT−, BMP2+); Values are expressed as the mean ± SD, *n* = 8. **c** Gla-Osteocalcin contents of primary calvarial osteoblast cultures in the absence and presence of 4-OHT at day 6 after BMP2 stimulation. *p < 0.005 versus *SMS1*^*f/f*^*;SMS2*^*−/−*^ (4-OHT+, BMP2+) and *ERT2-Cre;SMS1*^*f/f*^*;SMS2*^*−/−*^ (4-OHT−, BMP2+); Values are expressed as the mean ± SD, *n* = 6. **d** After treatment with and without 1 μM 4-OHT for 1 day, *SMS1*^*f/f*^*;SMS2*^*−/−*^ and *ERT2-Cre;SMS1* ^*f/f*^*;SMS2*^*−/−*^ mice calvarial osteoblasts were cultured with and without 100 ng/ml BMP2 in α-MEM with 10% FBS containing 50 μg/ml ascorbic acid, 10 mM β-glycerophosphate for 4 days. *Col1a1*, *BSP* and *OPN* mRNA levels were normalized to GAPDH mRNA. Treatment with 4-OHT does not affect the differentiation of primary *SMS1*^*f/f*^*;SMS2*^*−/−*^ calvarial osteoblasts. *p < 0.005 versus *SMS1*^*f/f*^*;SMS2*^*−/−*^ (4-OHT+, BMP2+) and *ERT2-Cre;SMS1*^*f/f*^*;SMS2*^*−/−*^ (4-OHT−, BMP2+); Values are expressed as the mean ± SD, n = 8. **e** Decreased bone nodule formation in the 4-OHT-induced *SMS1* inactivation. Representative images of nodule mineralization stained with Alizarin red. *SMS1*^*f/f*^*;SMS2*^*−/−*^ and *ERT2-Cre;SMS1*^*f/f*^*;SMS2*^*−/−*^ mice calvarial osteoblasts were treated with and without 1 μM 4-OHT for 1 day, and cultured with and without 100 ng/ml BMP2. On day 17, the cells were stained with Alizarin red (upper). Quantification of the staining is shown (lower). *p < 0.005 versus *SMS1*^*f/f*^*;SMS2*^*−/−*^ (4-OHT+, BMP2+) and *ERT2-Cre;SMS1*^*f/f*^*;SMS2*^*−/−*^ (4-OHT−, BMP2+); Values are expressed as the mean ± SD, n = 8

To gain further insight into how SMS1 affects the activity of primary neonatal osteoblasts, we investigated whether loss of SMS1 activity affects the in vitro mineralization capacity of cultured osteoblasts. Because bone formation of *Sp7-Cre;SMS1*^*f/f*^*;SMS2*^*−/−*^ mice was poor, sufficient osteoblast numbers could not be obtained from *Sp7-Cre;SMS1*^*f/f*^*;SMS2*^*−/−*^ calvarial bone. We also established a system to induce ablation of SMS1 in osteoblasts from *ERT2-Cre;SMS1* ^*f/f*^*;SMS2*^*−/−*^ mice in vitro. ERT2-Cre mice were crossed to enable tracking and visualization of Cre-mediated inactivation of SMS1. By crossing *SMS1*^*f/f*^*;SMS2*^*−/−*^ mice with those mice, tamoxifen-inducible SMS1-deficient *SMS2*^*−/−*^ mice (*ERT2-Cre;SMS1*^*f/f*^*;SMS2*^*−/−*^) were created that were able to provide sufficient neonatal calvarial osteoblasts; ERT2-Cre negative control calvarial osteoblasts were prepared from *SMS1*^*f/f*^*;SMS2*^*−/−*^ mice. An analysis by qRT-PCR showed that SMS1 expression was decreased in osteoblasts treated with 4-OHT (Fig. 4b). The above system was also used to determine whether induced inactivation of SMS1 affected osteoblast proliferation and differentiation. First, we examined the cell growth activity of osteoblasts as shown in the scheme in Fig. 4a. After a 4-day treatment with 4-OHT, the cell number was slightly increased. Addition of FGF2 increased the cell number further in both *SMS1*^*f/f*^*;SMS2*^*−/−*^ and *ERT2-Cre;SMS1*^*f/f*^*;SMS2*^*−/−*^osteoblasts (Fig. 4c). These results indicated that the ability to stimulate the growth of osteoblasts with FGF2 was not affected by deletion of SMS1. Next, we examined the differentiation of osteoblasts (Fig. 5a). ALP activity and Gla-OCN synthesis, both of which are early osteoblast differentiation markers, were then analyzed (Fig. 5b, c). ALP activity and Gla-OCN synthesis were significantly increased 4 days after stimulation with BMP2 in *SMS1*^*f/f*^*;SMS2*^*−/−*^ and *ERT2-Cre;SMS1* ^*f/f*^*;SMS2*^*−/−*^ osteoblasts without 4-OHT treatment. In contrast, there was no BMP2-stimulated increase in either ALP activity or Gla-OCN synthesis in 4-OHT-treated *ERT2-Cre;SMS1* ^*f/f*^*;SMS2*^*−/−*^osteoblasts. An analysis by qRT-PCR showed that addition of BMP2 significantly increased COL1A1, BSP, and OPN mRNA expression levels in *SMS1*^*f/f*^*;SMS2*^*−/−*^ and *ERT2-Cre;SMS1* ^*f/f*^*;SMS2*^*−/−*^ osteoblasts without 4-OHT. However, 4-OHT-treated *ERT2-Cre;SMS1*^*f/f*^*;SMS2*^*−/−*^ osteoblasts showed reduced COL1A1, BSP, and OPN mRNA expression after BMP2 treatment (Fig. 5d). In mineralization assays, quantification of Alizarin Red S staining was used for terminal differentiation of osteoblasts. Osteoblasts were treated in the same manner as for ALP assays, except that osteoblasts were analyzed 14 days after stimulation with BMP2. After stimulating with BMP2, *SMS1*^*f/f*^*;SMS2*^*−/−*^ and *ERT2-Cre;SMS1*^*f/f*^*;SMS2*^*−/−*^ osteoblasts without 4-OHT became significantly mineralized. In contrast, *ERT2-Cre;SMS1*^*f/f*^*;SMS2*^*−/−*^ osteoblasts treated with 4-OHT were less mineralized than those without 4-OHT treatment (Fig. 5e). These data suggest that inactivation of SMS1 inhibits the BMP2-induced differentiation of osteoblasts.

### Inactivation of SMS1 inhibits BMP2-induced phosphorylation of Smad1/5/8 and p38 in primary osteoblasts

**Fig. 6:**
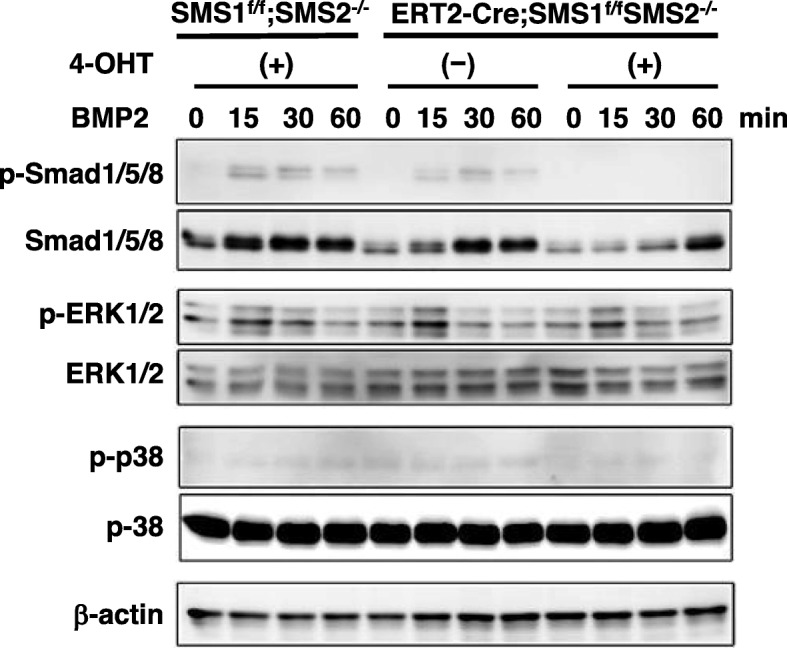
Inactivation of SMS1 inhibited BMP2-induced Smad1/5/8 and p38 phosphorylation. The cellular phosphorylation levels of Smad1/5/8, ERK1/2, and p38 were determined by Western blot analysis of femoral osteoblasts from *SMS1*^*f/f*^*;SMS2*^*−/−*^ and *ERT2-Cre;SMS1*^*f/f*^*;SMS2*^*−/−*^ mice. After treatment with and without 1 μM 4-OHT for 1 day, calvarial osteoblasts were serum starved for 12 h before BMP2 (50 ng/ml) stimulation for the indicated times, and then immunoblotted with antibodies specific to phospho-Smad1/5/8, phospho-ERK1/2, and phospho-p38

To gain further insight into the potential mechanism by which inactivation of SMS1 inhibits BMP2-induced osteoblast differentiation, we next examined the effect of SMS1 deficiency on BMP2 signaling pathways [i.e., Smad and mitogen-activated protein kinase (MAPK)] in primary osteoblasts. BMP2 stimulation rapidly induced phosphorylation of intracellular Smad1/5/8, Erk1/2, and p38, key downstream molecules of BMP2 signal transduction. When *ERT2-Cre;SMS1*^*f/f*^*;SMS2*^*−/−*^ calvarial osteoblasts were treated with 4-OHT, BMP2-induced Smad1/5/8 phosphorylation was significantly inhibited. Next, we examined the effect of inactivation of SMS1 on MAPK signaling. We showed that BMP2-induced p38 phosphorylation was mildly inhibited but Erk1/2 phosphorylation was unaffected by inactivation of SMS1 (Fig. 6). These results showed impaired BMP2 signaling in SMS1-deficient osteoblasts, suggesting that SMS1 functions to regulate osteoblast activity in cooperation with BMP2 signaling.

## Discussion

SM is generated by SMS1 and SMS2. Function of both SMS1 and SMS2 in different organs were studied by SMS1 and SMS2 knockout mice. However, it remains unknown whether SMS1 and/or SMS2 has a direct effect in bone formation. The goals of the current study are to characterize the skeletal phenotype of mice that lack SMS1 and/or SMS2 and to investigate the local roles SMS1 and SMS2 play in osteoblasts. In this study, we generated conditional knockout mice by crossing Sp7 promoter-driven Cre-recombinase-expressing mice and mice homozygous for the floxed SMS1 gene. SP7 (osterix) is required for an important step in the differentiation of pre-osteoblasts into fully functional osteoblasts (Nakashima and de Crombrugghe 2003). Using the SP7-Cre drive to conditionally inactivate SMS1 in pre-osteoblasts and block SMS1 function in these cells, we found a low-bone-mass phenotype with decreased trabecular bone and inhibited osteoblast differentiation in this study. Although SP7-Cre mice suffer delayed growth of cortical bone and low body weight in the early stage of development, these effects have largely disappeared by 12 weeks of age when the mice are skeletally mature (Davey et al. 2012). Wang et al. reported that there were severe intramembranous bone-formation defects in SP7-Cre mice during the early postnatal stage but that mineralization gradually improved during development and had become almost completely normal by 3 weeks of age (Wang et al. 2015). Additionally, we used control SP7-Cre mice in our study when appropriate.

It is clear that SMS1 is important for whole-body health, especially considering that SMS1-deficient mice exhibit moderate neonatal lethality (Yano et al. 2011). To our knowledge, SMS1 conditional knockout in osteoblasts has not been investigated in Sp7 promoter-driven Cre mice. In this study, we have demonstrated for the first time that SMS1 conditional knockout (*Sp7-Cre;SMS1*^*f/f*^*;SMS2*^*−/−*^) causes bone development defects, while SMS2 knockout (*SMS1*^*f/f*^*;SMS2*^*−/−*^) does not. After birth, there is a difference in body weight between Sp7-Cre-positive mice and Sp7-Cre-negative mice. However, SP7-Cre-positive littermate controls have delayed growth characterized by lower body weight until 10 weeks of age, but this delay in growth is overcome by the time adulthood is reached at 12 weeks. We found that the volume of cortical and trabecular bone in our Sp7-controlled conditional SMS1 knockout mice was decreased, indicating that their overall weight loss and growth retardation may primarily result from bone abnormalities in adulthood. On the other hand, SMS2 knockout mice are characterized neither by reduced body weight nor a reduced volume of cortical and trabecular bone. These results imply that SMS2 does not affect bone development. However, it is unknown whether SMS1 has a direct functional role in bone formation. In the present study, we have addressed this question by demonstrating the effect of bone-specific deletion of SMS1 on postnatal skeletal development and thus establishing the function of SMS1 to regulate the development of osteoblasts. We found that the skeletal phenotype of E15.5, E17.5, newborn, and 15-week-old Sp7-controlled conditional SMS1 knockout mice exhibit reduced mineralization of calvaria and long limb bones in the early stages of ossification and reduced trabecular number, trabecular bone volume, and cortical bone thickness in vivo. On the other hand, SMS2^−/−^ and SP7-Cre-positive littermate controls showed no reduction of trabecular number, trabecular bone volume, or cortical bone thickness in vivo. These results imply that Sp7-controlled conditional SMS1 knockout mice have decreased bone mass, which results in bone defects during pre- and postnatal growth.

Cortical bone thickness was reduced in affected *Sp7-Cre;SMS1*^*f/f*^*;SMS2*^*−/−*^ mice compared to control mice. Although there was no increased osteoid in adult *SMS1*^*f/f*^*;SMS2*^*−/−*^, *Sp7-Cre;SMS1*^*+/+*^*;SMS2*^*+/−*^ mice, the amount of osteoid was mildly increased in adult *Sp7-Cre;SMS1*^*f/f*^*;SMS2*^*−/−*^ mice. However, Von Kossa stained sections from *Sp7-Cre;SMS1*^*f/f*^*;SMS2*^*−/−*^ mice did not demonstrate characteristic features of hypophosphatemic rickets including increased width of the unmineralized epiphyseal growth plate and increased trabecular bone volume in the proximal metaphysis. The growth-plate regions demonstrated normal cartilage calcification in the hypertrophic zone and at the epiphyseal end of the growth plate in *Sp7-Cre;SMS1*^*f/f*^*;SMS2*^*−/−*^ mice. In Von Kossa-stained sections, we consistently observed a tendency for greater osteoid volume in *Sp7-Cre;SMS1*^*f/f*^*;SMS2*^*−/−*^ mice, and histomorphometric analysis revealed that this was statistically significant. These results imply that the osteoblast differentiation process was affected in *Sp7-Cre;SMS1*^*f/f*^*;SMS2*^*−/−*^ mice.

In bone, RANKL is expressed in osteoblasts and their precursors, while RANK is present on the cell-surface membranes of osteoclasts and their progenitors (Nagy and Penninger 2015). It is therefore a marker of bone resorption. In this study, in femoral bone of *Sp7-Cre;SMS1*^*f/f*^*;SMS2*^*−/−*^ mice, mature ALP (osteoblast marker) expression level was significantly downregulated, while RANKL (osteoblast functional marker) and RANK (osteoclast marker) expression levels were slightly downregulated. RANK and RANKL were slightly less compared to control mice, showing that the resorption process in *Sp7-Cre;SMS1*^*f/f*^*;SMS2*^*−/−*^ mice was limited or retarded. OPG, the marker of bone protection against erosion, showed a mild downregulation compared to control mice. This trend of OPG is correlated with the decrease of RANK and RANKL in *Sp7-Cre;SMS1*^*f/f*^*;SMS2*^*−/−*^ mice. The RANKL/OPG ratio is considered a comprehensive indicator of prevailing activity of bone remodeling (Canciani et al. 2017). The RANKL/OPG ratio is negative not only in *Sp7-Cre;SMS1*^*f/f*^*;SMS2*^*−/−*^ but in control mice as well. It might be that bone apposition is prevalent compared to resorption. Furthermore, ALP activity in cultured *Sp7-Cre;SMS1*^*f/f*^*;SMS2*^*−/−*^ osteoblasts was significantly decreased, while TRAP activity was unaltered. TRAP staining of femur sections revealed that the number of TRAP-positive multinucleated osteoclasts was unchanged in *Sp7-Cre;SMS1*^*f/f*^*;SMS2*^*−/−*^ mice; this finding was confirmed by histomorphometric analysis. Osteoclast-related parameters, the percentage of bone surface covered by mature osteoclasts (Oc.S/BS), and the number of mature osteoclasts (N.Oc/B.Pm) were comparable to controls. To determine the role of SMS1 in osteoclast development, bone-marrow-derived monocyte/macrophage precursor cells (BMMs) isolated from C57BL/6, *SMS1*^*f/f*^*;SMS2*^*−/−*^, and *Sp7-Cre;SMS1*^*f/f*^*;SMS2*^*−/−*^ mice were treated with mM-CSF and mRANKL in vitro*.* Osteoclast differentiation was not affected in *Sp7-Cre;SMS1*^*f/f*^*;SMS2*^*−/−*^ BMMs as indicated by TRAP staining and activity (data not shown). These data suggest that decreased trabecular bone volume in *Sp7-Cre;SMS1*^*f/f*^*;SMS2*^*−/−*^ mice is not due to increased bone resorption. The histomorphometric analysis of the present study revealed a significantly increased number of osteoids in trabecular bone, with a slightly increased number of osteoclasts, in *Sp7-Cre;SMS1*^*f/f*^*;SMS2*^*−/−*^ mice, but not in *SMS1*^*f/f*^*;SMS2*^*−/−*^ mice or SP7-Cre-positive littermate control mice. These results imply that arrested osteoblast development contributes, at least in part, to reduced bone formation observed in Sp7-controlled conditional SMS1 knockout mice.

Primary neonatal osteoblast culture is a well-established cell-culture system to analyze in vitro osteoblast differentiation. The observation that Sp7-controlled conditional SMS1 knock-out caused significantly compromised osteoblast mineralization in vivo raised the question of whether it would affect osteoblast differentiation and mineralization in vitro. Wang et al. showed that both early and terminal differentiation of primary calvarial osteoblasts isolated from neonatal SP7-Cre mice were comparable to those of control cells (Wang et al. 2015). In the present study, to investigate the mechanisms underlying bone defects in *Sp7-Cre;SMS1*^*f/f*^*;SMS2*^*−/−*^ mice, we focused on the response to BMP2-induced osteoblast differentiation and mineralization in primary osteoblasts. We found that after BMP2 stimulation 4-OHT-treated SMS1-inactivated osteoblasts were significantly decreased in ALP activity, BSP and OPN expression, and Gla-OCN synthesis. These results suggest that impaired bone formation is not caused by the inhibition of osteoblast proliferation but rather by suppression of early and late stages of osteoblast differentiation.

Next, we found suppression of phospho-Smad1/5/8 and phospho-p38, but not phospho-ERK1/2, in 4-OHT-treated SMS1-inactivated osteoblasts after BMP2 stimulation. Smad family members play major roles in transforming signaling in the growth-factor-β superfamily (Macias et al. 2015; Retting et al. 2009). BMP2-induced phosphorylation of Smad1/5/8 cooperates with Runx2 (Leong et al. 2009; Schroeder et al. 2005), and Runx2 is an essential osteoblast-related gene regulator that promotes expression of ALP (Lemonnier et al. 2004). Therefore, activation of Smad1/5/8 is required for osteoblast differentiation. MAPKs, such as p38 and ERK1/2, were also activated by BMP2 in our study and have been documented to participate in the differentiation-inducing effects of BMP2 in MC3T3 and primary osteoblasts (Egwuagu 2009). Moreover, MAPK signaling influences the development of osteoblast differentiation (Xiao et al. 2002a, b), and FGF signaling is known to regulate osteoblast proliferation via MAPK pathways (Ornitz and Itoh 2015). P38 signaling has been shown to play a major role in endochondral bone development (Stanton et al. 2004).

Recently, it has been reported that BMP2 regulates SMPD3 expression in osteoblasts via p38 mitogen-activated protein kinase and its downstream transcription factors. However, the major role of BMP2 signaling involving SMAD1/5/8 may not have a direct effect on the transcriptional regulation of SMPD3 (Manickam et al. 2019). In the present study, in which ERT2-Cre;SMS1^f/f^;SMS2^−/−^ osteoblasts were treated with 4-OHT, BMP2-induced phosphorylation of both Smad1/5/8 and p38 was inhibited. Previous reports have shown that the amount of sphingomyelin species was decreased, whereas the amounts of ceramide species and glycosphingolipid were increased in SMS1-KO islets, suggesting that accumulated ceramide species were alternatively metabolized into glycosphingolipid species. However, there was no change of expression of SMPD3 mRNA in SMS1-KO islets (Yano et al. 2011). Considering these, SMS1 ablation may not be a direct regulator of SMPD3 expression, although sphingolipid metabolism may regulate BMP2-induced osteoblast maturation. In the present study, 4-OHT treatment affected differentiation in response to BMP2 in *ERT2-Cre;SMS1* ^*f/f*^*;SMS2*^*−/−*^ primary osteoblasts. These findings demonstrate an in vivo interaction between SMS1 and BMP2 in bone and suggest that SMS1 regulates osteoblast development in neonatal and postnatal bone formation through cooperation with BMP2 signaling. Our data identifying Smad1/5/8 and p38 as regulators of SMS1 expression imply that SMS1 may act as a downstream effector of Smad1/5/8 and p38. However, further studies are required to understand how various transcription factors cross talk to the BMPs in SMS1-regulated bone formation.

## Conclusion

The results of our current study suggest that knocking out *SMS1* in the SP7-expressing osteoprecursors impairs bone development and retards growth. Both of these effects are associated with the regulation of SMS1 in the development of osteoblasts. Further studies are needed to understand how various transcription factors cross-talk to regulate SMS1 expression in bone formation.

## Data Availability

All data produced or analyzed during this study are included in this published article.

## Abbreviations

4-OHT: 4-hydroxytamoxifen
ALP: Alkaline phosphatase
BMMs: Bone-marrow-derived monocyte/macrophage precursor cells
BMP: bone morphogenetic protein
BSP: Integrin binding sialoprotein
COLIA1: Collagen type I α1
CreERT2: Cre-recombinase estrogen receptor
DMP1: dentin matrix acidic phosphoprotein 1
FGF: fibroblast growth factor
GAPDH: Glyceraldehyde-3-phosphate dehydrogenase
OCN: Osteocalcin
OPG: Osteoprotegerin
OPN: Secreted phosphoprotein 1
PHEX: phosphate regulating endopeptidase homolog, X-linked
RANK: Receptor activator of nuclear factor κB
RANKL: Tumor necrosis factor ligand superfamily, member 11
Runx2: runt-related transcription factor-2
SM: sphingomyelin
SMS: sphingomyelin synthase
